# A Bone-Protective Role for IFN-γ? Evidence from Genetic Association and Osteoblast Functional Assays in Postmenopausal Osteoporosis

**DOI:** 10.3390/ijms27125548

**Published:** 2026-06-19

**Authors:** Camilla Albertina Dantas de Lima, Anna Paula Oliveira Souza, Maria Aparecida Barreto Lopes Seabra, Werbson Lima Guaraná, Bianca Maria Ribeiro de Oliveira, Sergio Crovella, Alexandre Domingues Barbosa, Jaqueline de Azevêdo Silva, Paula Sandrin-Garcia

**Affiliations:** 1 Department of Oceanography, Federal University of Pernambuco (UFPE), Recife 50670-901, PE, Brazil; camilla.lima@ufpe.br; 2 Laboratory of Immunopathology Keizo Asami (LIKA), Federal University of Pernambuco (UFPE), Recife 50670-901, PE, Brazil; werbson.guarana@ufpe.br (W.L.G.); bianca.maria@ufpe.br (B.M.R.d.O.);; 3 Biomedical Engineering Laboratory, Federal University of Pernambuco (UFPE), Recife 50670-901, PE, Brazil; 4 Department of Medicinal Chemistry, College of Pharmacy, University of Florida, Gainesville, FL 32611, USA; maseabra@ufl.edu; 5 Department of Genetics, Federal University of Pernambuco (UFPE), Recife 50670-901, PE, Brazil; 6 Rheumatology Division, Clinical Hospital of Federal University of Pernambuco, Recife 50670-901, PE, Brazil

**Keywords:** SNV, rs2069705, bone environment, protein expression

## Abstract

Osteoporosis (OP) is a complex disease in which several immune-related genes have been identified as contributing to susceptibility and disease progression. Despite efforts to achieve functional validation, many of these genes, such as interferon-gamma (*IFNG*), remain the subject of unresolved mechanisms. The present study aimed to examine whether the *IFNG* -1616 (G>A, rs2069705) polymorphism was associated with postmenopausal OP. A total of 251 OP patients and 115 healthy controls were genotyped to assess the association between the *IFNG* -1616 (G>A, rs2069705) polymorphism and osteoporosis. To further investigate the biological role of IFN-γ in bone metabolism, human SaOs-2 osteosarcoma cells were treated with recombinant IFN-γ (2 and 100 U/mL), and calcification and cell viability were evaluated using Alizarin Red staining and the MTT assay, respectively. We found that the *IFNG* rs2069705 G allele was associated with an increased risk of OP (OR = 1.45, 95% CI = 1.03–2.05, *p* = 0.03). Furthermore, serum IFN-γ levels did not differ significantly between genotype groups. In SaOs-2 cells, IFN-γ (2 U/mL) significantly increased viability (*p* = 0.017) and enhanced calcification in a dose-dependent manner. The *IFNG* rs2069705 G allele may confer susceptibility to postmenopausal OP. IFN-γ promotes osteoblast viability and mineralization at low concentrations, suggesting a potential anabolic role that warrants further investigation in human primary osteoblasts.

## 1. Introduction

Osteoporosis (OP) is a multifactorial metabolic bone disorder with a strong genetic component, accounting for approximately 50–85% of cases [1,2]. Like other complex traits, many genes with small effects contribute to the overall phenotype, including variations in bone mineral density (BMD), fracture susceptibility, bone turnover dynamics, and skeletal architecture [3,4]. A landmark Genome-Wide Association Study (GWAS) conducted in 2008 reinforced the functional interplay between the skeletal and immune systems, supporting the osteoimmunology framework initially proposed by Arron and Choi (2000) [5]. This evidence highlighted the role of cytokines and their signaling pathways in regulating BMD, fracture risk, and osteoporotic mechanisms [6,7].

Despite significant advances in high-throughput technologies that have led to the identification of novel candidate genes and numerous polymorphisms associated with OP, the precise biological functions of several cytokines involved in bone remodeling remain incompletely characterized [8,9,10]. Interferon-γ (IFN-γ) exemplifies this complexity, as its effects on bone tissue appear to be highly context-dependent, exhibiting both anabolic and catabolic properties depending on the cellular and molecular environment [11,12].

IFN-γ has already been reported to influence the survival, activation, and differentiation of osteoclasts by promoting Tumor Necrosis Factor receptor-associated factor 6 (TRAF6) degradation and blocking the RANK/RANKL pathway [12]. Conversely, previous studies have shown that IFN-γ promotes osteoclastogenesis by inducing specific cytokines and activating T cells [5,13,14]. Despite findings from GWAS [7] and proteomics [15] studies on the immune system and bone remodeling, only a few functional studies of *IFNG* polymorphisms in osteoporosis have been conducted to date. A previous study conducted by our group analyzing the same polymorphism demonstrated that the GG genotype of *IFNG* (G>A; rs2069705) was associated with both increased bone mineral density (BMD) at the femoral neck and decreased BMD at the total hip [16]. Furthermore, a study conducted in older adults with frailty syndrome demonstrated an association between the heterozygous genotype and susceptibility to the condition [17]. In addition, another study evaluating this polymorphism in patients with rheumatoid arthritis demonstrated that both the G allele and the GG genotype are associated with disease susceptibility [18]. Similarly, in a study involving primary Sjögren’s syndrome (pSS) [19], the G allele was also associated with disease susceptibility. The authors proposed that the rs2069705 single nucleotide variation (SNV) in the *IFNG* gene may play a central role in BMD variations and pSS susceptibility by enhancing IFN-γ transcription, subsequently activating the JAK/STAT1 signaling pathway [16,19]. Thus, this study assessed the role of IFN-γ by examining the promoter (SNV) *IFNG* -1616 (G>A; rs2069705) and its influence on serum cytokine levels and OP susceptibility. Additionally, the effects of this cytokine on bone mineralization were evaluated using the SaOs-2 osteosarcoma cell line, a validated in vitro model for mineralization that replicates human osteoblast cytokine expression [20].

## 2. Results

### 2.1. Studied the Population’s Characteristics

A total of 251 women (mean age ± SD: 62.15 ± 12.04 years) were classified as osteoporotic, whereas 115 participants (61.22 ± 5.13 years) were included as healthy controls based on the WHO diagnostic criteria for osteoporosis [21]. The clinical and demographic profile of the osteoporotic group is presented in Table 1.

### 2.2. Distributions of IFNG Genotypes and Risk of Osteoporosis

The distribution of genotypes and alleles for the *IFNG* -1616 (G>A; rs2069705) variant across all analyzed groups is summarized in Table 2. Genotype frequencies observed in both osteoporosis patients and healthy control subjects were consistent with the Hardy–Weinberg equilibrium.

Significant differences in the allele distribution of *IFNG* rs2069705 were observed between individuals with osteoporosis and healthy controls. The G allele was more frequent among patients, suggesting an association with increased osteoporosis risk (OR = 1.45, 95% CI: 1.03–2.05; *p* = 0.03).

Regarding genotype distribution, the GG genotype was more prevalent in the osteoporosis group than in controls (22.4% vs. 14.6%). However, statistical significance was not consistently observed across all genetic models. Only the dominant model indicated a borderline association with disease susceptibility (OR = 1.62, 95% CI: 1.00–2.65; *p* = 0.053), as detailed in Table 2.

### 2.3. Serum Levels of IFN-γ in OP Patients and Healthy Controls

Regarding IFN-γ detection, 52.5% of individuals in the control group presented measurable levels (>0 pg/mL), whereas this proportion was lower among patients with osteoporosis (32.35%). Among individuals with detectable cytokine levels, median serum concentrations [range] were higher in controls (2.12 [1.47–9.52] pg/mL) compared to the osteoporotic group (1.03 [0.82–12.27] pg/mL) (Table 3).

Within the osteoporosis group, individuals carrying the *IFNG* rs2069705 GG genotype exhibited the highest IFN-γ levels (2.60 [2.00–12.27] pg/mL), followed by AA (1.80 [1.28–2.32] pg/mL) and GA genotypes (0.97 [0.61–5.81] pg/mL) (Table 3). In contrast, a distinct distribution pattern was observed in the control group, where the AA genotype was associated with the highest cytokine levels (5.51 [1.86–7.36] pg/mL), followed by GA (3.64 [1.47–7.69] pg/mL) and GG genotypes (2.78 [1.56–9.42] pg/mL). Despite these variations in IFN-γ concentrations between groups and across genotypes, no statistically significant differences were identified (*p* > 0.05).

### 2.4. SaOs-2 Calcification Analysis

**Figure 1:**
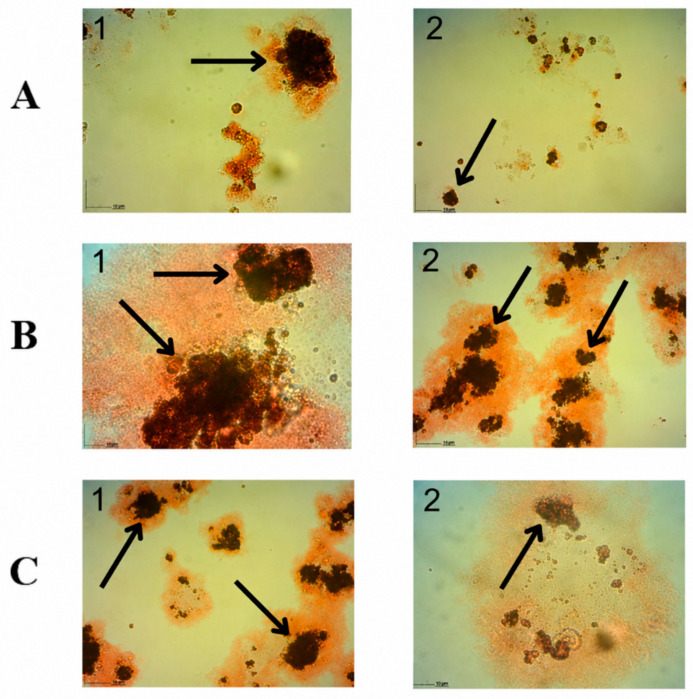
Effect of IFN-γ on SaOs-2 cell calcification. Cells were stimulated with IFN-γ for 24 h, and calcification was assessed by Alizarin Red staining. Representative images of (**A**) unstimulated control, (**B**) 2 U/mL IFN-γ, and (**C**) 100 U/mL IFN-γ. The numbers 1 and 2 represent magnifications of 400× and 100×, respectively. Calcified areas are indicated by arrows.

Calcification of stimulated or unstimulated cells was measured by Alizarin Red staining (Figure 1). IFN-γ increased calcification in vitro at both doses compared to unstimulated controls. Among the stimulated group, 2 U/mL IFN-γ showed higher calcification levels than 100 U/mL IFN-γ (69.14% and 19.15%, respectively, compared to unstimulated controls). Due to sample size limitations in the Alizarin Red quantification, these results are presented qualitatively and were not subjected to formal statistical testing.

### 2.5. Viability Assay

**Figure 2:**
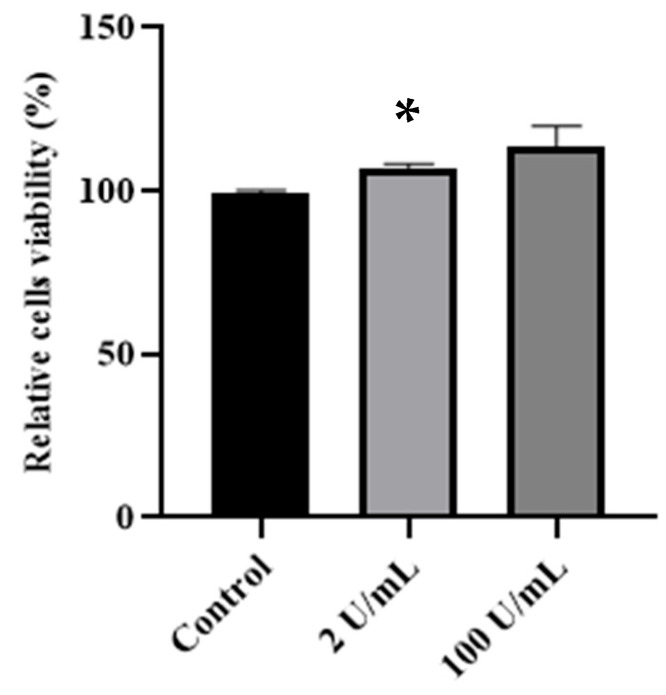
Effect of IFN-γ on SaOs-2 cell viability. Cells were stimulated with IFN-γ for 24 h, and viability was assessed by MTT assay. Data are presented as mean ± SD of three independent experiments. * *p* = 0.017 compared to unstimulated control (one-way ANOVA with Tukey’s post-test).

SaOs-2 cell viability was assessed using an MTT assay, and results showed that, compared to the unstimulated control, 2 U/mL and 100 U/mL of IFN-γ increased cell viability by 7.23% and 13.61%, respectively (Figure 2). However, only 2 U/mL showed a significant increase compared to the unstimulated control group (*p* = 0.017).

## 3. Discussion

The influence of interferon-gamma on osteoporosis and the bone microenvironment has been acknowledged in previous studies [11,12]; however, to our knowledge, this is the first study to report the impact of the *IFNG* -1616 (G>A, rs2069705) polymorphism in OP patients. Similarly, few studies have examined the effects of IFN-γ on osteoblasts [22,23,24]. Our findings show that the *IFNG* G allele is associated with increased susceptibility to OP. Additionally, low-concentration IFN-γ stimulation improved osteoblast-like cell calcification and viability, indicating a potential anabolic effect on human bone cells.

The *IFNG* -1616 (G>A, rs2069705) SNV is located in the promoter region of the *IFNG* gene, and the A variant has been previously associated with lower IFN-γ levels [9,25,26]. Although the G allele was associated with OP susceptibility in our study, no differences in *IFNG* expression levels were observed among genotypes. Similarly, investigations into infectious and autoimmune diseases have shown no association between the IFNG -1616 (G>A, rs2069705) polymorphism and *IFNG* mRNA expression levels. [26,27].

Our findings show that IFN-γ stimulation promoted calcification and viability in SaOs-2 cells, with the anabolic effect observed only at the lowest concentration tested (2 U/mL). The higher dose (100 U/mL) showed no significant effect on viability. This dose-dependent pattern aligns with Duque et al. (2011) [28], who reported that IFN-γ enhanced bone formation in ovariectomized mice and suggested that modulating the IFN-γ signaling pathway could improve bone strength. Similarly, Zhang et al. (2015) [29] found an association between IFN-γ and reduced bone resorption in postmenopausal women. However, the dual role of IFN-γ in bone homeostasis is well recognized [11,12]. While some studies highlight its direct anti-resorptive action through inhibition of the RANK/RANKL pathway and osteoclast formation, others describe an indirect pro-resorptive effect via T cell activation and induction of cytokines such as TNF-α and RANKL [30,31,32].

Importantly, most of these previous findings come from studies on osteoclasts or mouse models, with limited research on IFN-γ effects on human osteoblast-lineage cells. Among the few studies using osteoblast-like cells, Wimbauer et al. (2012) [33] reported that IFN-γ, when combined with 2-methoxyestradiol, exhibited anti-proliferative effects in osteosarcoma cells, in contrast to our observation of increased viability and calcification. These differences may be due to variations in experimental conditions, including cell type (SaOs-2 versus other osteosarcoma lines), concentration ranges, and the lack of additional agents in our model. Overall, our results highlight the context-dependent nature of IFN-γ activity in bone and suggest that low concentrations may have anabolic effects on human osteoblast-like cells, whereas higher concentrations may not.

Several mechanisms may explain the anabolic effects of IFN-γ on osteoblast-like cells observed in our in vitro assays. Ibarra Urizar et al. (2016) [34] demonstrated that IFN-γ (50 U/mL) stimulates bone morphogenetic protein 2 (BMP-2) expression, a key regulator of osteoblast differentiation and function [35,36,37]. Additionally, IFN-γ has been shown to synergize with IL-1 to induce nitric oxide (NO) production via endothelial nitric oxide synthase (eNOS), which may contribute to increased bone formation and decreased resorption [38,39,40]. In line with our findings, Duque et al. (2011) [28] reported dose-dependent anabolic effects of IFN-γ in ovariectomized mice, with lower concentrations promoting bone formation and higher concentrations potentially leading to bone loss [28,41,42]. Collectively, these observations suggest that IFN-γ effects on bone depend heavily on concentration, cell type, and experimental model, with human osteoblast-like cells responding differently from murine systems or osteoclast-focused models [13,43].

Finally, this study has several limitations. Although differentiation protocols were used to encourage osteoblast-like mineralization, the SaOs-2 cell line does not fully replicate the biological behavior of primary human osteoblasts; therefore, findings from this model should be interpreted with caution. Additionally, the strict inclusion criteria for the control group—excluding individuals with osteopenia, inflammatory or autoimmune diseases, cancer, and use of anti-inflammatory or other rheumatic medications—limited participant eligibility and resulted in a relatively small control group. Future functional studies with primary human osteoblasts and larger healthy control groups are needed to better understand the role of IFN-γ in bone metabolism. Another limitation is that only a single SNV within the *IFNG* promoter region was evaluated, which precluded haplotype and linkage disequilibrium analyses. Future studies investigating additional polymorphisms in linkage disequilibrium with the SNV under investigation may provide a more comprehensive understanding of the genetic regulation of IFN-γ in osteoporosis.

## 4. Materials and Methods

### 4.1. Subjects

In the present study, 251 patients diagnosed with postmenopausal osteoporosis and 115 healthy women from the Division of Rheumatology at the Clinical Hospital of the Federal University of Pernambuco, Brazil, were analyzed. All women underwent BMD measurements by dual-energy X-ray absorptiometry (Hologic or Lunar) to diagnose OP at the lumbar spine (vertebrae L1–L4), the femoral neck (FN), and the total femur (TF). Women classified as osteoporotic according to World Health Organization criteria were included in the patient group, while those classified as healthy were included in the healthy control group [21].

The groups were evaluated for secondary diseases according to a health assessment questionnaire. Patients with osteopenia, inflammatory disease, autoimmune diseases, cancer, under the use of anti-inflammatories, and other rheumatic diseases were excluded from this study.

The sample size was calculated using G*Power 3.1.9.7. A theoretically large effect size was found (0.5 for the Chi-square analysis, 0.50 for the *t*-test, and 0.4 for the ANOVA test). The significance level (α) was set at 0.05, with a desired power of 0.8. The sample size was calculated for analyses involving patient samples, including comparisons of genotype frequencies, and cytokine level assessments. The parameters used are consistent with previous studies [44,45,46].

This study was approved by the Research Ethics Committee of the Center for Health Sciences, Federal University of Pernambuco (CEP/CCS/UFPE no.513/11), and all participants signed a written informed consent form.

### 4.2. DNA Isolation and Determination of IFNG Genotypes

Genomic DNA was isolated from human whole blood cells using the rapid salting-out method [47]. Genotyping of the extracted DNA was performed with the Real-Time PCR ABI 7500 detection system (Applied Biosystems, Foster City, CA, USA) using a specific fluorogenic probe for *IFNG* rs2069705 (C_15944115_20, TaqMan Probes, Applied Biosystems, Foster City, CA, USA).

### 4.3. In Vitro Assays

The SaOs-2 cell line was derived from an osteosarcoma of an 11-year-old Caucasian female patient from the public tissue bank at the Federal University of Rio de Janeiro (UFRJ) (Cell Line: Saos-2, BCRJ Code: 0217, ATCC: HTB-85) and was maintained in a humidified incubator at 37 °C with 5% CO_2_. For experiments, cells were cultured in 6-well plates and maintained for 15 days in hMSC Osteogenic Differentiation BulletKit™ Medium (Lonza, Basel, Basel-Stadt, Switzerland) to induce calcification. The media changes were performed every 3 days.

Following this calcifying process, confluent SaOs-2 cells were seeded at 2 × 10^5^ cells/well and then treated with or without recombinant human IFN-γ (Peprotech, Hamburg, Germany). The maximum (100 U/mL) and minimum (2 U/mL) concentrations were used, following a previous analysis with a range of 0.5 to 100 IU/µL, expanding to 10 initial test concentrations (0.5; 1; 2; 2.5; 5; 10; 20; 30; 40; 50; 60; 70; 80; 90 and 100) in the MTT assays. From this analysis, we selected the minimum and maximum concentrations with statistically significant effects on cell viability, and these are the concentrations used in the study (2 and 100 IU/uL). After IFN-γ stimulation, calcification and viability were assessed after 24 h, while cytokine levels were measured at 6, 8, and 24 h.

### 4.4. Alizarin Red Staining and Quantification

Calcified SaOs-2 cells were plated in 24-well plates and incubated at 37 °C with 5% CO_2_ for 24 h, with or without IFN-γ (2 U/mL and 100 U/mL). Cells were stained with Alizarin Red (pH 4.2, Sigma cat# A5533) (Sigma Chemical Co., St. Louis, MO, USA) for 20 min, then washed five times for five minutes with deionized water. A 5% Cetylpyridinium Chloride (Sigma cat# C0732) (Sigma Chemical Co., St. Louis, MO, USA) solution was used to dissolve the alizarin red that bound to calcium deposits, and the concentration was measured using a spectrophotometer at λ = 570 nm (Varioskan Flash—Thermo Scientific, Rockford, IL, USA).

### 4.5. Cell Viability Assay

Calcified cells were stimulated for 24 h in the presence of IFN-γ in 24-well plates (2 U/mL and 100 U/mL). Mitochondrial activity was measured using methyl-tetrazolium bromide (MTT) according to the manufacturer’s instructions (Sigma Chemical Co., St. Louis, MO, USA). An MTT reagent solution (Sigma Chemical Co., St. Louis, MO, USA) (5 mg/mL) was added and incubated at 37 °C with 5% CO_2_, and the color was observed to change to brown. After 3 h, the medium was carefully removed, and DMSO was used to dissolve all crystals. The plate was incubated for 5 min at 37 °C and 5% CO_2,_ and the absorbance was measured at λ = 550 nm with a microplate reader (Dynex Technologies, Chantilly, VA, USA).

### 4.6. Measurement of IFN-γ Serum Levels from OP Patients and Th1/Th2 Cytokines from Cell Culture

IFN-γ serum levels from 37 OP patients and 34 healthy women, as well as Th1 and Th2 cytokines (IL-2, IL-4, IL-6, IL-10, TNF-α) supernatants from the SaOs-2 cell line, were measured using a Cytometric Bead Array (CBA) with the CBA Human Th1/Th2 Kit, following the manufacturer’s protocol (BD Biosciences, San Jose, CA, USA). All data were obtained using a Becton Dickinson FACScalibur flow cytometer (BD Biosciences, San Jose, CA, USA), and analyses were performed with BD CBA software (Version 2.0) (BD Biosciences, San Jose, CA, USA). The results are presented as mean fluorescence intensity (MFI).

### 4.7. Statistical Analysis

The SNPStats tool was used to calculate the Hardy–Weinberg equilibrium [48]. The comparison of allele and genotype frequencies between healthy controls and OP patients was performed using the chi-square test. Student’s *t*-test and analysis of variance (ANOVA) with Tukey’s test as a post hoc test were used to compare mean values. These analyses were conducted with SPSS software version 18.0, and the graphs were created using GraphPad Prism software version 8.0 (GraphPad Software, La Jolla, CA, USA). A *p*-value less than 0.05 was considered statistically significant.

## 5. Conclusions

In conclusion, our findings reinforce the relevance of osteoimmunological pathways in postmenopausal osteoporosis and provide new evidence supporting the involvement of *IFNG* genetic variation in disease susceptibility. The *IFNG* rs2069705 G allele was associated with an increased risk of osteoporosis, while patients also exhibited significantly higher *IFNG* mRNA expression levels in PBMCs, suggesting an altered immune profile in the disease. Although no direct association was observed between rs2069705 genotypes and circulating IFN-γ or *IFNG* transcription levels, the in vitro assays demonstrated that low concentrations of IFN-γ enhanced osteoblast-like cell viability and mineralization, supporting a potential anabolic and dose-dependent role of this cytokine in bone metabolism. These results highlight the complex and context-dependent nature of IFN-γ activity in bone homeostasis, where immune signaling can exert both protective and deleterious effects depending on the cellular environment and cytokine concentration. Collectively, our data contribute to a better understanding of the immunogenetic mechanisms underlying osteoporosis and suggest that IFN-γ-related pathways may represent promising targets for future investigations. Nevertheless, additional studies using primary human osteoblasts, functional promoter assays, broader genetic approaches, and *IFNG* quantification in stimulated and unstimulated PBMCs are required to clarify the molecular mechanisms linking *IFNG* variants, cytokine regulation, and bone remodeling in osteoporosis.

## Figures and Tables

**Table 1:** Clinical and demographic characteristics of postmenopausal osteoporosis patients. Data are presented as mean ± SD for continuous variables and as percentages for categorical variables.

**Characteristics**	**OP Patients (n = 251)**	
Age (years ± SD)	62.15 ± 12.04	
Age at menarche (years ± SD)	13.59 ± 1.90	
Age at menopause (years ± SD)	45.66 ± 6.81	
Disease duration (years ± SD)	1.64 ± 2.30	
BMI (kg/m^2^ ± SD)	25.90 ± 4.38	
**Characteristics**	**Presence (%)**	**Absence (%)**
Smoking	8.28	91.72
Alcoholism	5.94	94.06
Fragility facture	13.58	86.42
Family osteoporosis	38.58	61.41

OP = Osteoporosis; BMI = Body mass index.

**Table 2:** Distribution of genotypes and allele frequencies of *IFNG* rs2069705 SNVs among OP patients and healthy controls.

Model	Genotypes	OP n (%)	Controls n (%)	OR (95% CI)	*p*
Codominant	AA	65 (26.5)	38 (36.9)	1	0.082
	GA	125 (51)	50 (48.5)	0.68 (0.41–1.15)	
	GG	55 (22.4)	15 (14.6)	2.13 (1.05–4.30)	
Dominant	AA	65 (26.5)	38 (36.9)	1	0.053
	GA + GG	180 (73.5)	65 (63.1)	1.62 (1.00–2.65)	
Recessive	GG	55 (22.4%)	15 (14.6%)	1	0.094
	AA + GA	190 (77.5%)	88 (85.4%)	0.60 (0.31–1.11)	
Overdominant	GA	125 (51)	50 (48.5)	1	
	AA + GG	120 (49)	53 (51.5)	0.91 (0.57–1.45)	0.067
	**Alleles**				
	A	255 (52)	126 (61)	1	
	G	235 (48)	80 (39)	1.45 (1.03 –2.05)	0.03 *

OP = Osteoporosis; *p* = *p* value; OR = odds ratio; CI = confidence interval; * *p* < 0.05 was considered statistically significant.

**Table 3:** IFN-γ protein expression according to *IFNG* rs2069705 SNVs among OP patients and healthy control groups.

Genotypes	OP pg/mL (Range)	*p*	Controls pg/mL (Range)	*p*
AA	1.80 [1.28–2.32]	0.39	5.51 [1.86–7.36]	0.80
GA	0.97 [0.61–5.81]		3.64 [1.47–7.69]	
GG	2.60 [2.00–12.27]		2.78 [1.56–9.42]	

OP = Osteoporosis; *p* = *p* value.

## Data Availability

The original contributions presented in this study are included in the article. Further inquiries can be directed at the corresponding author.
